# The roles of nuclear focal adhesion kinase (FAK) on Cancer: a focused review

**DOI:** 10.1186/s13046-019-1265-1

**Published:** 2019-06-11

**Authors:** Jin Zhou, Qian Yi, Liling Tang

**Affiliations:** 1 Key Laboratory of Biorheological Science and Technology, Ministry of Education, College of Bioengineering, Chongqing University, Chongqing, 400044 China; 2 Department of Physiology, School of Basic Medical Sciences, Southwest Medical University, Luzhou, 646000 Sichuan China

**Keywords:** Nuclear FAK, Cancer, Transcription factors, Gene expression, Inhibitors

## Abstract

FAK is a tyrosine kinase overexpressed in cancer cells and plays an important role in the progression of tumors to a malignant phenotype. Except for its typical role as a cytoplasmic kinase downstream of integrin and growth factor receptor signaling, related studies have shown new aspects of the roles of FAK in the nucleus. FAK can promote p53 degradation through ubiquitination, leading to cancer cell growth and proliferation. FAK can also regulate GATA4 and IL-33 expression, resulting in reduced inflammatory responses and immune escape. These findings establish a new model of FAK from the cytoplasm to the nucleus. Activated FAK binds to transcription factors and regulates gene expression. Inactive FAK synergizes with different E3 ligases to promote the turnover of transcription factors by enhancing ubiquitination. In the tumor microenvironment, nuclear FAK can regulate the formation of new blood vessels, affecting the tumor blood supply. This article reviews the roles of nuclear FAK in regulating gene expression. In addition, the use of FAK inhibitors to target nuclear FAK functions will also be emphasized.

## Background

Numerous studies on the potential link between FAK and different kinds of cancer have gradually revealed the biological mechanisms by which FAK promotes the development and progression of cancer [1]. FAK is a tyrosine kinase with a molecular weight of 125kD, playing a vital role in cellular communication, especially in cell signaling systems [2]. Wang et al. [3] revealed that increased mRNA levels, protein levels, and the activation of FAK were positively associated with cancer metastasis and invasion and frequently inversely correlated with better clinical cancer sample results in the detection of human cancer samples. Relevant studies have found that FAK was overexpressed and/or over-phosphorylated in multiple cancer cells, responsible for cell migration [4], survival [5], proliferation [6], and adhesion [7]. In addition, FAK is strongly associated with the occurrence and development of tumors [2, 8] and regarded as a functional protein in the cytoplasm, typically functioning in a kinase-dependent manner [9]. Firstly, FAK receives different extracellular signals coming from cell-surface transmembrane receptors including integrins, cytokines, growth factors, and G protein-coupled receptors. After that, FAK activates and triggers subsequent signaling cascades in a variety of cellular activities [10, 11]. FAK can also participate in the signal transduction process in tumor vessel, mediating the vessel permeability [12–14]. The FERM domain of FAK can combine with the cytoplasmic region of vascular endothelial calcium mucin. It is important for cell-cell adhesive junctional structures, an integral part of keeping vascular integrity [15]. Furthermore, FAK is essential for maintaining vascular functions in tumor angiogenesis. Lees et al. [16] found that FAK recovered the vascular leakage defect through the activation of kinase domain. And it is a fact that cytokines induce vascular growth factor expression by the FAK signaling pathway. For example, via Src-FAK-STAT3 signaling, IL-6 induces VEGF-C expressions [17]. As a result, FAK kinase activity is required for tumor growth [18], angiogenesis [17], and vascular permeability [19]. These show that FAK is a typical multifunctional protein which integrates and transduces signals into cancer cells via integrin or growth factor receptors. Tumor stem cells are few tumor cells which are present in malignant cells and believed to be the source of cancer cells. They have the ability to proliferate, self-renew and generate heterogeneous tumor cells, maintaining the vitality of the tumor cell population [20, 21]. Yoon et al. [22] found that FAK promoted cancer stem cells (CSCs) renewal and drug resistance by functioning in survival signaling. For example, FAK and the extracellular signal-regulated kinase (ERK1/2) pathway are involved in the regulation of growth and metastasis of liver cancer stem cells (LCSCs) [23]. The use of the anticancer drug salinomycin inhibited the activity of FAK and ERK1/2, resulting in the increased stiffness of LCSCs [24]. Another study has shown that changes in the stiffness of living cells might affect numerous cellular physiological activities [25]. FAK can affect the growth of LCSCs through this mechanism of the regulation of cell stiffness. Cheng et al. [26] targeted HIC1 and RassF1A methylation, induced the transformation of mesenchymal stem cells (MSCs) and the cell stiffness was lost. It is suggested that Tumor cells are softer than normal cells, mainly due to loss of cytoskeletal support [27, 28]. And the loss of stiffness can represent a phenotype of tumor development which facilitates cancer cell migration and adapts to other tissues [29, 30]. Taken together, these results indicate that FAK is closely related to biological behaviors such as survival, migration, invasion, and proliferation of CSCs. Based on those findings, FAK can be regarded as a target for cancer therapy.

Actually, investigators have found that FAK was also functional in the nucleus [31]. FAK can enter the nucleus and regulates gene expression to influence tumorigenesis [32]. In the nucleus, activated FAK binds to transcription factors to regulate gene expression. Inactive FAK synergizes with different E3 ligases to promote the turnover of transcription factors [33]. FAK affects tumor survival and growth by altering the transcription [34]. In this review, some regulation modes of nuclear FAK are discussed. We focus on nuclear FAK regulating gene expression in different cancer cells. FAK regulates gene expression by affecting the expression of transcription factors. Furthermore, we emphasize that nuclear FAK also has an important role in the study of cancer, which is positively related to the occurrence and development of tumors.

## FAK can shuttle between cytoplasm and nucleus

### The structure of FAK

**Fig. 1:**
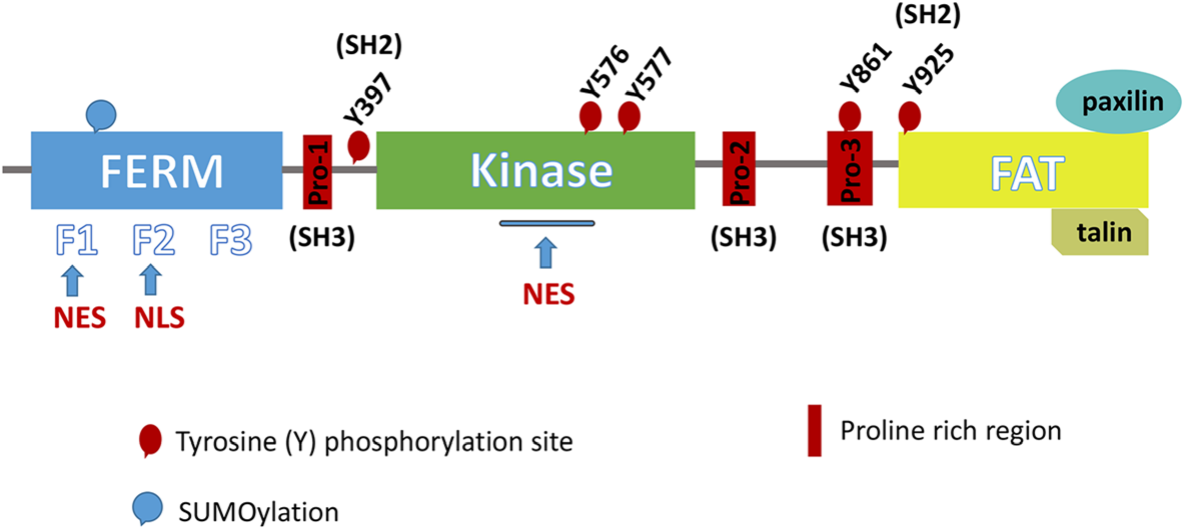
Schematic structure of FAK. The N-terminal FERM is shown in blue, containing three subdomains of F1, F2, and F3. The SUMOylation is located in the FERM domain. NES1 is located in the F1 leaf and NLS is located in the F2 leaf. The kinase domain is shown in green containing NES2 and the FAT domain is shown in yellow responsible for interacting with primary adhesion plaque components such as Paxillin and Talin. There are proline-rich regions between the domains and they are binding sites of Src homology 3 (SH3). Significantly, there are five important tyrosine phosphorylation sites. Among them, Y397 and Y925 are binding sites of Src homology 2 (SH2)

In humans, FAK is composed of the N-terminal containing the FERM domain, the central kinase domain, and the C-terminal with the FAT domain (Fig. 1) [35, 36]. The FERM domain consists of approximately 300 amino acid residues, binding directly to the intracellular portion of the transmembrane protein receptors [37]. The kinase domain refers to the 390–650 amino acid region which is highly conserved. It has at least 6 tyrosine phosphorylation sites [38, 39], which is the key to FAK signaling. The FAT domain is responsible for interacting with primary adhesion plaque components such as Paxillin [40], Talin [41], Grab2 [42], Rgnef/p190RhoGef [43], and vascular endothelial growth factor receptor 3 (VEGFR3) [44]. Notably, FAK contains binding sides for more than 50 proteins, permitting FAK to function as a kinase and molecular scaffolds [45].

It was reported that FAK had nuclear export signals (NES) in the kinase domain and nuclear localization signals (NLS) in the F2 lobe of the FERM domain [31, 46], which led to the shuttle of FAK between the focal adhesions (FAs) and the nucleus. Further analysis of NLS and NES showed that bare alkaline residue clusters in the surface of NLS were K190, K191, K216, K218, R221, and K222 and the NES was composed of a leucine-rich amino acid sequence [34]. And it was showed that FAK had two NES sequences actually [36, 1, 47]. One was NES1 located in the F1 lobe and the other was NES2 located in the kinase domain. Although both of them are conservative in the evolutionary process, only NES2 has complete biological nuclear export activities [46, 1]. In addition, apart from facilitating phosphatidylinositol 4,5-bisphosphate (PIP2) lipid binding [48], the residues of F2 lobe (KAKTLR) can also function as a nuclear localization sequence [49]. Post-translational modifications of FAK include binding protein inhibitors of activated STAT1 (PIAS1) to the FERM domain of FAK and the addition of a small ubiquitin-related modifier (SUMO) to the Lys152 residue [50]. SUMOylation is always in connection with nuclear import signals and the SUMO of nuclear FAK is related to FAK activation [51]. Under the action of stimulation or biomolecules, such as chemical and/or genetic stress, FAK can occur nuclear recruitment [52, 53]. The nuclear export sequences and nuclear localization sequences are crucial for the entry of FAK into the nucleus and the process of FAK entering the nucleus requires physiological or chemical induction.

### The nuclear localization of FAK

FAK can shuttle between the cytoplasm and the nucleus. Membrane anchors such as FAs may immobilize FAK at the adhesion sites, keeping FAK in the cytoplasm. But FAs such as Paxillin and Zyxin families can also migrate to the nucleus [40, 54, 55]. Although neither protein links DNA directly, they can serve as coreceptors of transcription factors. Thus the interaction of FAK with those proteins may jointly regulate nuclear translocation of integrin adherent structures [56]. For example, the ectopic expression of PIAS1 promotes FAK c-terminal protein cleavage, focal adhesion maturation, and FAK nuclear localization in non-small cell lung cancer (NSCLC) cells. Moreover, nuclear p125 FAK was found to be significantly phosphorylated at the Y861 site by specifically expressing the FAK fragments and the amino-terminal domain FAK fragments could regulate the nuclear localization and apoptotic in endothelial cells [57]. Constanzo et al. [58] found that nuclear FAK activity promoted the survival and progression of NSCLC by increasing cellular-extracellular matrix (ECM) interaction and DNA repair regulation. In a related study, neural cell adhesion molecule (NCAM) induces FAK activation and the nuclear import of the C-terminal fragment and N-terminal fragment of FAK [59]. Moreover, it is speculated that the localization of FAK in the nucleus facilitates the formation of FAs complexes by inhibiting autophagy, thereby triggering cell proliferation [47, 58]. It suggests that the nuclear recruitment of FAK requires the participation of other proteins, such as NCAM and the activation of FAK can also affect the nuclear recruitment and regulate DNA repair.

Scientists have found the molecular mechanisms which stimulated FAK from the cytoplasm into the nucleus [31]. Firstly, Lim et al. [34] treated cancer cells with an apoptotic inducer of staurosporine which led to FAK nuclear accumulation. Treatment of myocytes with H_2_O_2_ can lead to oxidative stress, promote FAK nuclear localization, and induce myocyte differentiation [60]. These indicate that stress signals promote FAK migration from the cytoplasm to the nucleus. Secondly, the x-linked apoptotic protein inhibitor (XIAP) promotes FAK localization in the FAs, which enhances the phosphorylation of FAK at pY576. Meanwhile, XIAP knockout reduces the phosphorylation of FAK, promoting the shear stress-induced translocation of FAK to the nucleus [61]. The desorption of cells from the matrix may increase the free FAK available in the cytoplasm, leading to FAK nuclear localization by detachment from focal adhesion [31]. Similarly, Aoto et al. [62] mutated the proline-rich region of Pyk2. They mutated proline 859 to alanine, prevented the binding of SH3-containing proteins, and promoted the detachment of Pyk2 from FAs and the nuclear localization of Pyk2. Therefore, the decellularization of cells from the matrix leads to FAK nuclear localization. Thirdly, Lim et al. [63] found that kinase depletion (KD) FAK cells had stronger FAK nuclear localization signals than wild-type (WT) FAK. Studies have shown that FAK inhibitors such as PF-562,271, PF-271, Pfizer, could significantly increase the nuclear localization of FAK [64, 65]. This result is consistent with the previous result of kinase depletion. It is speculated that activated FAK may be localized to the cytoplasm or adhesion preferentially, instead of the nucleus [34]. Inhibitors can modify FAK, inhibit FAK activity and induce it to enter the nucleus. Therefore, FAK inhibition promotes FAK nuclear localization. The three different ways suggest that stress signals, the desorption of cells from the matrix, and the inhibition of FAK may induce FAK into the nucleus.

## FAK functions in the nucleus

### The function of FAK in regulating p53 pathway

FAK enters the nucleus from the FAs site via FERM NLS. After that, FAK binds to the N-terminal transactivation domain of p53 (Table 1) through its N-terminal FERM domain, decreases the transcriptional activity of p53 and inhibits p53 to activate its downstream gene transcription [67]. A further research found that the binding site of p53 for interaction with FAK was a 7-amino-acid site in the proline-rich region in the N-terminal domain [68]. In this progress, the inactivation of p53 requires F1 leaf interacting with p53, KAKTLR-mediated nuclear localization, F3 leaf connecting murine double minute2 (Mdm2) and proteasome degradation [31]. A Study has found that FAK negatively regulated p53 expression in mesothelioma cell lines and NF2 regulated the interaction of FAK–p53 and Mdm2–p53 [69]. The FERM domain mediates the transfer of FAK into the nucleus and binds to p53, which in turn binds to Mdm2, leading to the degradation of p53 through the ubiquitination pathway and inhibiting apoptosis. FAK promotes p53 transformation by enhancing Mdm2-dependent p53 ubiquitination [31, 82]. This mechanism implies that p53 ubiquitination and Mdm2-mediated proteasomal degradation keep p53 at a low level, promoting cell survival. At this point, this is one of the earliest kinase-independent roles, which requires only FAK’s scaffolding function.

**Table 1:** Proteins interacting with nuclear FAK

Protein	Outcome	Significant Findings
NCAM	FAK phosphorylation	NCAM induces the nuclear import of the C-terminal fragment and N-terminal fragment of FAK [59]
PIAS1	Post-translational modifications	PIAS1 binds to the FERM domain of FAK, affecting its post-translational modifications [51, 66]
MBD2	Dissociation from HDAC1	Activate the expression of myogenic proteins and other genes that promote muscle differentiation [60]
P53	Degradation	FAK inhibits p53 to activate its downstream gene transcription [67, 68]
Mdm2	P53 ubiquitination	P53 proteosomal degradation in the nucleus [31, 69]
NF2	Activation	NF2 regulates the interaction of FAK–p53 and Mdm2–p53 [69]
PTEN	Upregulation	P53 and PTEN tumor suppressors are co-inactivated in patients and cause cancer metastasis [70]
GATA4	Degradation	FAK inhabits GATA4 expression and reduces the inflammatory responses [58, 65, 71]
CHIP	GATA4 ubiquitination	The nuclear FAK interacts with GATA4 and the ubiquitin protein E3 ligase CHIP [58, 65, 72]
IL-33	Upregulation	IL-33 regulates CCL5 expression [73, 74]
VEGFR2	Upregulation	VEGFR2 promotes the formation of tumor neovascularization and tumor growth [75]
Sin3A	Activation	FAK participates in the regulation of Runx1 via Sin3A [76]
Runx1	Runx1 complex	The formation of the transcription factor Runx1 complex [77]
MEF2	Forms complex	FAK and MEF2 jointly regulate expression of Jun which is induced by load [78]
FIP200	FAK phosphorylation inhibition	FIP/FAK complex is associated with FAK inactivation after cell detachment [78]
EZH2	EZH2 phosphorylation	FAK affects the transcription and nuclear localization of EZH2 [79]
E3 ligase	Ubiquitination	Inactive FAK coordinates with different E3 ligases, promoting transcription factor turnover [65, 71]
Sam68	Activation	Sam68 binds to RNA and signaling molecules to regulate multiple signaling pathways [80]
NS	Activation	Active FAK protects the NS from proteasomal degradation [81]

Nuclear FAK can regulate p53-mediated cell behavior after binding to p53. The earlier studies have shown that almost 50% of tumors had mutations in p53 and p53 inactivation was a key step in tumorigenesis [83, 84]. P53 negatively affects the expression of many genes that promote cell senescence, cell apoptosis and cell death, such as p21, Gadd45, Cyclin G, Bax, Gml, and P2xm. At the same time, p53 inhibits the promoter activity of various cell cycle and anti-apoptotic genes, such as Cyclin, Bax/Bcl2, and IGF-BP3 [85]. For example, apolipoprotein B mRNA editing enzyme catalytic polypeptide-like (APOBEC) is an important source of tumorigenic mutations. Among them, APOBEC3B (A3B) expression is particularly relevant to tumors, such as tumor resistance [86]. Periyasamy et al. demonstrated a negative correlation between A3B expression and p53 expression in different cancer types, as p53 played a direct and critical role in inhibiting A3B expression [87]. The CXCR4 receptor and FAK are believed to regulate the aggressive cancer behavior [70]. Their expression is down-regulated by p53 tumor suppressor and phosphatase and tensin homolog deleted on chromosome ten (PTEN). And p53 and PTEN tumor suppressors are co-inactivated in patients and cause cancer metastasis [88]. A recent study has found the mechanism of stabilizing nuclear p53. The type I phosphatidylinositol phosphate kinase (PIPKI-α) and its product phosphatidylinositol 4,5-bisphosphate (PtdIns(4,5)P) can maintain the stability of nuclear p53. The combination of PIPKI-α and p53 produces PtdIns(4,5)P, which promotes the interaction of small heat shock proteins with p53, thereby stabilizing the nuclear p53 [89]. P53 can also participate in the nucleolar stress pathway [90]. The nuclear mitotic apparatus protein (NuMA) is present in the nucleolus. NuMA can be involved in DNA damage as well as p53-mediated growth arrest and apoptosis [91]. Nuclear FAK binds to p53, reduce p53 levels and regulate the p53 signaling pathway in a kinase-independent manner. And as a multifunctional transcription factor, p53 tumor suppressor proteins regulate cellular processes that affect proliferation, cell cycle checkpoints, and apoptosis.

### The function of FAK in regulating inflammation pathway

Inflammation can alter the expression of oncogenes and tumor suppressor genes to promote the transition of cells to malignant tumors. It is estimated that infectious diseases and chronic inflammation account for about 25% of carcinogenic factors [92]. For example, DNA damage associated with inflammation in cancer stem cells can lead to cancer development with invasive clinical features [93]. The ROS/RNS caused by inflammation not only damages DNA, but also damages other biological macromolecules such as proteins and lipids, leading to dysfunction [94]. These indicate that inflammation is also closely related to tumor development. Inflammatory factors such as TNF-α can promote the expression of inflammatory genes through mitogen activated protein kinases (MAPKs) cascade and NF-κB activation [95, 96]. Therefore, inhibiting MAPKs and/or NF-κB pathway may significantly reduce the expression of inflammatory genes [97]. Aulakh et al. [72] found that the inhibition of FAK expression may effectively inhibit vascular cell adhesion factor-1 (VCAM-1) expression. Interestingly, although the inhibition of FAK expression blocks VCAM-1, it does not affect NF-κB activation [10, 65]. In this process, the activation of MAPKs does not affect VCAM-1 expression and FAK inhibition can promote the expression of GATA4 transcription factors [98]. This is mediated by the function of the nuclear FAK scaffold that interacts with GATA4 and the ubiquitin protein E3 ligase chips. Kinase-inhibited FAK has new developments and anti-inflammatory effects in limiting VCAM-1 expression through the nuclear localization and the promotion of GATA4 conversion [58, 65, 71]. This suggests that anti-inflammatory effects can be provided by the nuclear-localized FAK inhibition and it is important that the expressions of FAK and inflammatory cytokine are independent of NF-κB activation.

At the same time, a research showed that FAK also induced the expression of inflammatory genes and the products of these genes inhibited the anti-tumor immunity in the microenvironment, leading to the immune escape of tumor [99]. Firstly, researchers found that FAK depletion or inhibition could lead to squamous cell carcinoma regression. Nuclear FAK induces the expression of immunosuppressive molecules cytokines and chemokines, forming an immunosuppressive microenvironment, leading to tumor escape [73]. These factors drive the depletion of CD8^+^ T cells and the recruitment of regulatory T cells (Tregs) [100], resulting in the depletion of antigen-induced cytotoxic CD8^+^ T cell activity that allows tumor growth [101]. Tumor invasiveness has a positive correlation with the number and size of nucleoli. In nucleoli, FAK binds a cancer stem cell marker riboflavin and protects it from stress-induced degradation. A further study revealed that nuclear FAK bound to the inflammatory factor IL-33 and regulated the expression of chemokine ligand 5 (CCL5) and growth stimulation expressed gene 2 (ST2) [74]. IL-33 binds to CD8 T cells, leading to tumor cells to escape the recognition of CD8 T cells [102]. Deletion of CCL5 reduces tumor-infiltrating Treg cells, resulting in regression of FAK-WT tumors [103]. ST2 is secreted into the tumor environment as a decoy receptor, resulting in competitive inhibition of IL-33/ST2 autocrine and paracrine signals [104]. Those studies demonstrate the roles of FAK in the nucleoli. FAK protects nucleocapsid proteins from proteasomal degradation that is essential for breast cancer growth. In general, FAK-IL33 regulation is similar to FAK-GATA4 regulation, indicating that nuclear FAK is actually a scaffold promoting transcription factor turnover and regulating of inflammatory factor expression.

### The potential roles of nuclear FAK

The formation of blood vessels plays an important role in the occurrence and development of tumors [105]. A study has confirmed that anti-tumor effects could be achieved by inhibiting neovascularization [106]. Targeting tumor vascular endothelial cells to inhibit tumor angiogenesis and block tumor blood supply has become a research hotspot for current anti-tumor. FAK is an indispensable protein in embryonic angiogenesis and regulates angiogenesis in kinase-independent and kinase-dependent manners. In endothelial cell (EC), FAK acts in a kinase-independent manner, regulating cell survival and barrier function. FAK deletion or inhibition of activity reduces EC proliferation and migration [107]. This suggests that FAK acts primarily as a kinase that regulates EC-mediated angiogenesis. Further mechanistic analysis revealed that FAK could regulate the expression of vascular endothelial growth factor receptor 2 (VEGFR2). Nuclear FAK is directly involved in the transcriptional regulation of VEGFR2 via the VEGFR2 promoter-associated RNA polymerase II complex [75, 65]. VEGFR2 is the central substance of angiogenesis. It can bind to VEGF-C and VEGF-D, regulating vascular endothelial cells and lymphatic endothelial cells, promoting lymphangiogenesis and blood vessel formation, and regulating lymphocyte migration. This shows that FAK is also important to maintain the tumor microenvironment. In addition, nuclear FAK can promote the formation of tumor neovascularization and tumor growth.

**Fig. 2:**
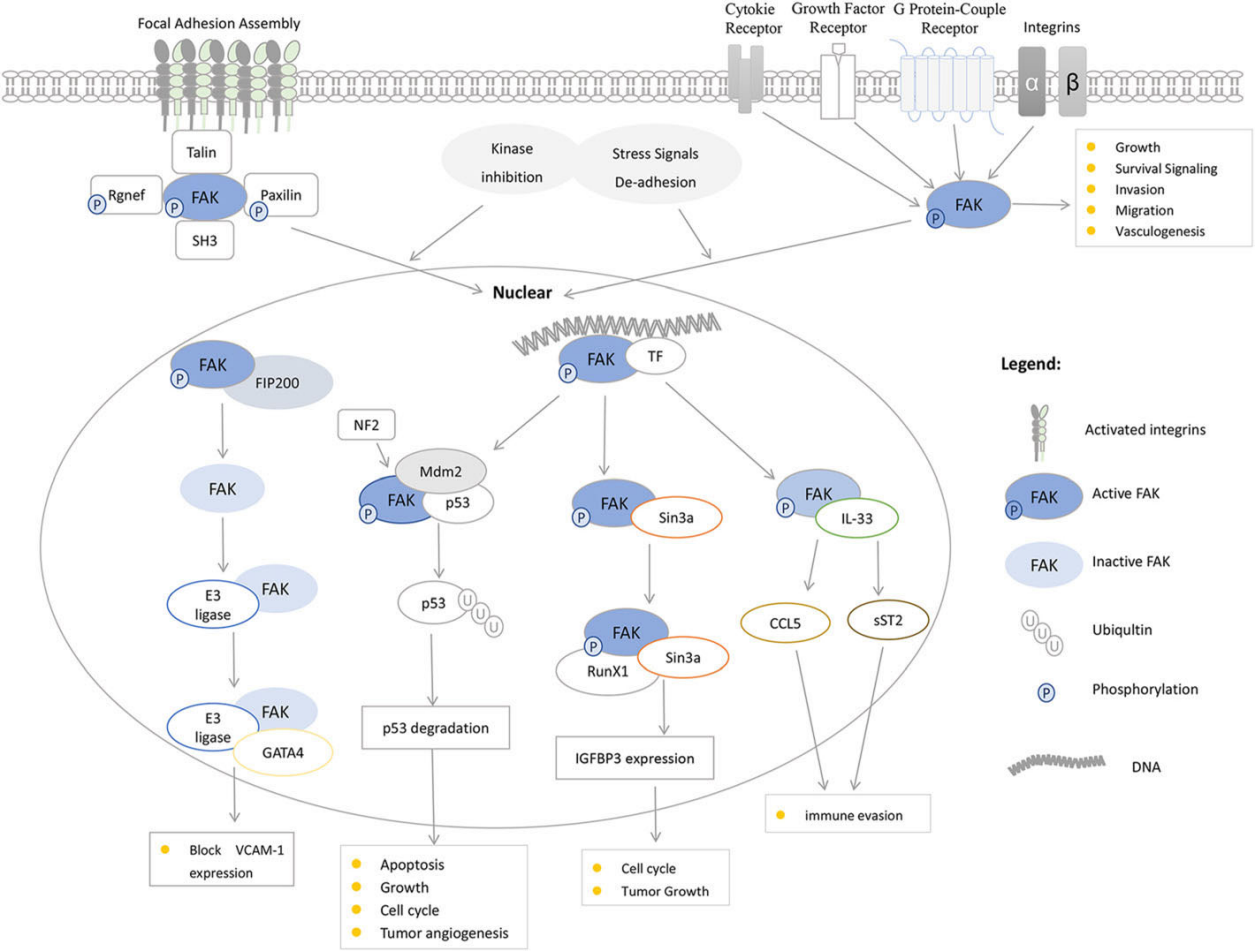
FAK functions in the nucleus. The activated FAK phosphorylates Rgnef and paxillin to promote focal adhesions assembly. In the cytoplasm, FAK regulates the development of cancer. Via the FERM structure, cell de-adhesion and/or kinase inhibition can promote FAK shuttle from cytoplasm to the nucleus. After entering the nucleus, active FAK binding to transcription factors (TFs) regulates cancer-related gene expression. Inactive FAK coordinates with different E3 ligases promoting turnover of TFs by enhancing ubiquitination. By regulating gene expression, nuclear FAK can be involved in the regulation of tumor angiogenesis, cell cycle, apoptosis, cell proliferation, immune escape, and tumor growth

The SIN3 transcriptional regulatory protein family member A (Sin3A) is a core component of a multiprotein transcriptional repressor complex [108, 109]. Nuclear FAK is involved in the regulation of the formation of the transcription factor Runx1 complex by interacting with Sin3A [76]. Runx1 regulates the expression of insulin-like growth factor binding protein 3 (IGFBP3). IGFBP3 is an extracellular secretory protein that binds to IGF and regulates IGF signaling. IGFBP3 also exhibits ligand-independent function in cultured mammalian cells. In cancer, IGFBP3 regulates cell cycle progression, affecting cell proliferation and tumor growth [110]. The roles of FAK in the nucleus are largely mediated by the FERM domain, and the FAT domain is also involved. It forms a complex with myocyte enhancer factor 2 (MEF2) transcription factors and upregulates transcriptional activity under mechanical stress [111]. Early evidence showed that FIP200 colocalized with nuclear FAK in focal adhesions. Furthermore, the FIP/FAK complex is associated with FAK inactivation after cell detachment [78]. Serrels et al. [73] found that FAK activity increases in squamous carcinoma cells compared to normal keratinocytes and thus FAK nuclear localization is related to cell transformation. And then a recent study has found that FAK affected the transcription and nuclear localization of zeste homolog 2 (EZH2) by regulating the transcriptional activities of p53 and E2F2/3 [79]. In high blood pressure, FAK and FAK-related non-kinase (FRNK) enter the nucleus. Protein kinase C (PKC) mediates the nuclear translocation of FAK and FRNK [112]. In the nucleus, FAK and FRNK can bind to different nuclear proteins, such as Src mitosis-associated protein 68 (Sam68) and fibrin, then targeting different nuclear regions [80]. Nucleolus is a non-membrane nuclear structure that regulates ribosome biogenesis and cell proliferation [113]. Proteins associated with nucleoli, such as nucleolar phosphoprotein B23 and nuclear stabilizing protein (NS), play an important role in genomic protection, ribosome synthesis, and stem cell proliferation [114, 115]. Tancioni et al. inhibited FAK activity and led to a decrease in proteasome-mediated NS levels. They found the mechanism of FAK in nucleoli by which active FAK protected the NS from proteasomal degradation and Akt-mTOR pathway regulated the stability of NS in breast cancer cells [81]. In general, activated FAK in the nucleus controls a variety of transcription factors leading to changes in gene regulation. Meanwhile, inactive FAK coordinates with different ubiquitin protein ligase E3 that promotes transcription factor turnover by enhancing ubiquitination (Fig. 2).

## FAK inhibitors

There are two important topics in the field of cancer research: one is tumor molecular imaging and the other is targeted molecular therapy. According to the consensus of many publications, FAK can be used as a promising target for anticancer therapies [116]. Currently, targeting FAK as a method of treating tumors mainly focuses on the use of drugs to inhibit its kinase activity and scaffold function. And reported FAK inhibitors are mainly small molecule inhibitors [1]. Small chemical molecules with good drug-forming properties can inhibit the phosphorylation of FAK and block the signal transduction through the cell membrane, thereby inhibiting the proliferation and spread of cancer cells [117]. Therefore, the research on FAK inhibitors is very promising. The pharmacodynamic activities of FAK inhibitors that have entered preclinical or clinical studies can be divided into two categories according to their mechanisms: ATP-dependent and ATP-independent [118]. The ATP-dependent FAK inhibitors can affect the binding of ATP to FAK and block FAK phosphorylation. The ATP-independent FAK inhibitors do not pass through the ATP binding site, but directly targets the FAK site, such as the FAK Y397 phosphorylation site [119]. Experimental results also showed that those small molecule FAK inhibitors could inhabit cell migration [3], survival [120], proliferation [121], and adhesion [122]. FAK inhibitors also can inhibit nuclear active FAK phosphorylation and regulate its related signaling pathways, such as the p53 signaling pathway, the inflammatory signaling pathway, the tumor angiogenesis-related pathway, and the immune escape signaling pathway. These pathways are closely related to tumor survival, migration, invasion, growth. For example, Dao et al. [123] found that 1,3,5-triazinic inhibitors of FAK could resist angiogenesis in HUVEC cells and have anticancer effects on various cancer cells. On this basis, they designed and synthesized a new compound containing a 1,2,4-triazine core as an inhibitor of FAK. And the compound can effectively inhibit the proliferation of U-87MG and HCT-116 cancer cells and exhibit a good anti-tumor effect [124]. In addition, Qu et al. [125] synthesized a class of FAK inhibitors, named Sul-DPPYs and it could effectively inhibit the activity of FAK and treat pancreatic cancer as a potent FAK inhibitor. Experiments have shown that treatment of NSCLC cells with CXCR4 and FAK inhibitors such as WZ811 and PF-573228 can inhibit their ability to migrate and invade [70, 88]. Inducing expression of p53 and p21 in ECs by down-regulating FAK may result in damage to angiogenesis and tumor growth [31]. What’s more, Roslin2 or 1-benzyl-15,3,5,7-tetraazetidine[3.3.1.1~3,7~] decane (R2) compounds disrupt FAK and p53 proteins that subsequently suppress tumor growth [85, 126]. In addition, small molecule inhibitors can inhibit FAK-mediated immune escape [73]. VS-4718 can inhibit the expression of immunosuppressive molecules such as IL-33 and CCL5, and reduce Tregs in the tumor environment [74]. Although inhibitors inhibit FAK phosphorylation, it is also possible to selectively induce nuclear localization. For example, PF-562,271 can block the phosphorylation of FAK at Y397 site and significant increase the nuclear localization of inactive FAK [64, 65]. The inactive FAK enters the nucleus and binds to E3 ligase to regulate the expression of transcription factors. And the mechanism of FAK kinase inhibitor targeting immunosuppressive may represent an effective immunomodulatory therapy. The development of FAK inhibitors is currently underway, and many inhibitors have shown therapeutic effects on cancer. Therefore, research on FAK inhibitors is also one of the research hotspots and it is also one of the directions for the development of anti-tumor drugs.

## Conclusion

Current researches on FAK focus on the roles of FAK in FAs. FAK is a cytoplasmic non-receptor protein tyrosine kinase that phosphorylates different targets in cells. FAK also has a very important position in cell signal transduction. It is the center of intracellular and extracellular signal transduction and mediates multiple signaling pathways. FAK can be used as a platform to participate in the assembly of protein complexes and a bridge to participate in the signal transduction between proteins. Similarly, FAK also plays an important role in tumor cell signal transduction, mediating the tumor progression to a malignant invasion phenotype. Through these kinase-dependent mechanisms, FAK can regulate biological behaviors of tumor cells such as adhesion, migration, invasion, proliferation and survival.

Since FAK has a nuclear export signal, a nuclear localization signal, and the SUMOylation in the FERM domain related to nuclear import signals. FAK can also enter the nucleus via biological mechanisms. Nuclear FAK controls various transcriptional networks such as the p53 signaling pathway, the inflammatory signaling pathway, the immune escape, and angiogenesis, influencing multiple cancer cell functions. The inhibition of nuclear FAK expression can affect the biological behavior of tumor cells such as aging, apoptosis and immune escape. However, the regulation mechanism of FAK in the nucleus remains to be further studied. For example, FAK regulates p21 cyclin-dependent kinase inhibitor gene expression in a kinase-dependent or kinase-independent manner, but how FAK regulates the expression of the p21 gene in two ways remains unclear. The molecular mechanism is still unclear. In tumor angiogenesis, whether FAK regulates the expression of related molecules through other pathways still needs to be studied. Furthermore, both FAK and p53 can participate in the regulation of nucleolar associated proteins expression in the nucleolus. But it is not clear whether they interact. Therefore, there are still many problems in this field that have not yet been solved. In the future, it is necessary to explore its molecular mechanisms, which is crucial for studying the occurrence and development of tumors. Furthermore, further study of the roles of nuclear FAK may uncover new mechanisms that promote tumor development.

## Data Availability

Not applicable

## Abbreviations

A3B: APOBEC3B
APOBEC: **A**polipoprotein B mRNA editing enzyme catalytic polypeptide-like
CCL5: Chemokine ligand 5
CSCs: Cancer stem cell
EC: Endothelial cell
ECM: Extracellular matrix
ERK1/2: Extracellular signal-regulated kinase
EZH2: Zeste homolog 2
FAK: Focal adhesion kinase
FAs: Focal adhesions
FAT: C-terminal
FRNK: FAK-related non-kinase
IGFBP3: Insulin-like growth factor binding protein 3
KAKTLR: FERM F2 lobe
KD: Kinase depletion
LCSCs: Liver cancer stem cells
MAPKs: Mitogen activated protein kinases
Mdm2: Murine double minute2
MEF2: Myocyte enhancer factor 2
MSCs: Mesenchymal stem cells
NCAM: Neural cell adhesion molecule
NES: Nuclear export signals
NLS: Nuclear localization signals
NS: Nuclear stabilizing protein
NSCLC: Non-small cell lung cancer
NuMA: Nuclear mitotic apparatus protein
PIAS1: Protein inhibitor of activated STAT1
PIP2: Phosphatidylinositol 4,5-biphosphate
PIPKI-α: Type I phosphatidylinositol phosphate kinase
PKC: Protein kinase C
PtdIns(4**,**5**)**P: Phosphatidylinositol 4,5-bisphosphate
PTEN: Tensin homolog deleted on chromosome ten
R2: 1-benzyl-15,3,5,7-tetraazetidine[3.3.1.1~3,7~] decane
Sam68: Src mitosis-associated protein 68
SH2: Src homology 2
SH3: Src homology 3
Sin3A: SIN3 transcriptional regulatory protein family member A
ST2: Growth stimulation expressed gene 2
SUMO: Small ubiquitin-related modifier
TF: Binding transcription factor
Tregs: Regulatory T cells
VCAM-1: Vascular cell adhesion factor-1
VEGFR2: Vascular endothelial growth factor receptor 2
VEGFR3: **V**ascular endothelial growth factor receptor 3
WT: Wild-type
XIAP: X-linked apoptotic protein inhibitor
